# *Bifidobacterium animalis* subsp. *lactis* GCL2505 modulates host energy metabolism via the short-chain fatty acid receptor GPR43

**DOI:** 10.1038/s41598-020-60984-6

**Published:** 2020-03-05

**Authors:** Hiroko Horiuchi, Kohei Kamikado, Ryo Aoki, Natsuki Suganuma, Tomohiko Nishijima, Akiho Nakatani, Ikuo Kimura

**Affiliations:** 1 Institute of Health Sciences, Ezaki Glico Co., Ltd., Osaka, 555-8502 Japan; 2 Department of Applied Biological Science, Graduate School of Agriculture, Tokyo University of Agriculture and Technology, Tokyo, 183-8509 Japan

**Keywords:** Fat metabolism, Metabolic syndrome, Endocrine system and metabolic diseases, Obesity, Nutritional supplements

## Abstract

Short-chain fatty acids (SCFAs), which are metabolites derived from the fermentation of dietary fibre by the gut microbiota, are important for host metabolic health. There is interest in probiotics for their beneficial effects on metabolic disorders, such as obesity, but the underlying mechanisms remain largely unknown. In this study, we evaluated whether *Bifidobacterium animalis* subsp. *lactis* GCL2505 (GCL2505), a probiotic strain capable of proliferating and increasing SCFA levels in the gut, exerts anti-metabolic syndrome effects via the SCFA receptor G protein-coupled receptor 43 (GPR43). A GCL2505 treatment suppressed body fat accumulation, improved glucose tolerance, and enhanced systemic fatty acid oxidation in high-fat diet (HFD)-fed wild type (WT) mice, whereas these effects were not observed in HFD-fed *Gpr43* knockout (*Gpr43−/−*) mice. Caecal and plasma acetate levels were elevated by GCL2505 in WT and *Gpr43−/−* mice, but the negative correlation between plasma acetate levels and body fat accumulation was observed only in WT mice. We further demonstrated that GCL2505 suppressed insulin signalling in the adipose tissue via GPR43. These results suggested that increases in SCFA levels in response to GCL2505 enhance host energy expenditure, which decreases fat accumulation via activated GPR43.

## Introduction

Metabolic syndrome, which has emerged as a worldwide epidemic and a major public health issue^1^, is primarily characterised by obesity, insulin resistance, hyperlipidaemia, and hypertension. Recent studies have suggested that the gut microbiota has important physiological functions affecting host energy metabolism as well as the development of obesity, insulin resistance, and other hallmarks of the metabolic syndrome^2,3^. Changes in gut microbiota, such as decreases in the abundance of beneficial bacteria—for example, short-chain fatty acid (SCFA)-producing bacteria—and increases in the abundance of pro-inflammatory/pathogenic bacteria, are associated with the development of host metabolic abnormalities^4,5^. These observations suggest that modulating the gut microbiota is an effective approach for treating obesity and metabolic syndrome.

SCFAs are end products resulting from the fermentation of dietary fibre by gut microbiota. Specifically, SCFAs are saturated aliphatic organic acids consisting of one to six carbons, of which acetate (C2), propionate (C3), and butyrate (C4) are the most abundant (≥95%)^6^. Additionally, 95% of the produced SCFAs are rapidly absorbed in the caecum and large intestine, whereas the remaining 5% are excreted in faeces^7^. Although a substantial proportion of SCFAs is used as an energy source, SCFAs also activate specific G protein-coupled receptors (GPRs), namely GPR41 and GPR43^8^. Of these, GPR41 is activated equally by propionate and butyrate, whereas GPR43 is more responsive to acetate and propionate than to butyrate^9^.

SCFAs help regulate lipid and glucose metabolism, which has important implications for energy homeostasis^6^. We previously reported that acetate suppresses insulin signalling in adipocytes, thereby inhibiting the accumulation of fat induced by a high-fat diet (HFD) as well as promoting the metabolism of unincorporated lipids and glucose in other tissues^10^. However, these effects are not observed in *Gpr43* knockout (*Gpr43−/−*) mice, suggesting that SCFA-activated GPR43 is important for the storage of fat in white adipose tissue and the metabolism of lipids and glucose in other tissues.

Probiotics are live microorganisms that have been shown in controlled human studies to confer a health benefit to the host when administered in adequate amounts^11^. Although several probiotic strains and mixtures have been reported to improve metabolic syndrome^12^, the exact underlying mechanisms of individual strains remain relatively uncharacterized. *Bifidobacterium animalis* subsp. *lactis* GCL2505 (GCL2505), which was originally isolated from the faeces of a healthy adult, is a probiotic strain capable of proliferating in the gut^13,14^. We previously revealed that GCL2505 exerts anti-metabolic syndrome effects, such as improved glucose tolerance and the suppression of visceral fat accumulation^15^. In a clinical study, we also determined that the daily consumption of fermented milk containing GCL2505 decreases the amount of abdominal visceral fat^16^. Our earlier findings imply that the enhanced production of gut acetate is probably critical for the anti-metabolic syndrome effects of GCL2505. To elucidate the involvement of increased SCFA abundance in the anti-metabolic syndrome effects of GCL2505, we assessed the effects of GCL2505 on metabolic parameters and SCFA levels in *Gpr43−/−* and wild type (WT) mice.

## Results

### GCL2505 treatment suppressed body fat accumulation by promoting systemic fatty acid and glucose metabolism

**Figure 1:**
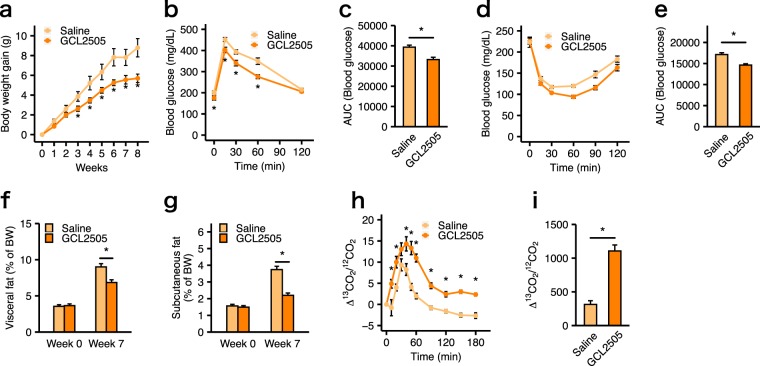
*Bifidobacterium animalis* subsp. *lactis* GCL2505 (GCL2505) treatments suppress body weight gain and improve metabolic parameters in high-fat diet-fed WT mice. (**a**) Body weight gain. (**b,c**) Blood glucose curve and the area under the curve (AUC) after an oral glucose challenge (2 g/kg body weight) in mice treated with GCL2505 or saline for 7 weeks. (**d,e**) Blood glucose curve and the AUC after an intraperitoneal injection of insulin (1 mU/g body weight) in mice treated with GCL2505 or saline for 7 weeks. (**f,g**) Computed tomography (CT)-estimated proportions of visceral and subcutaneous fat weight to body weight (BW) after 7-week probiotic treatments. Breath ^13^CO_2_:^12^CO_2_ isotope ratio expressed as delta over baseline (**h**) and the AUC (**i**) after an oral gavage of ^13^C-labelled potassium palmitate (60 μmol/kg body weight) in mice treated with GCL2505 or saline for 6 weeks. Data are presented as the mean ± SEM. Data were analysed with Welch’s *t*-test (*P < 0.05) (**a,c,e,f,g,h,i**) or two-way repeated-measurement ANOVA with the Bonferroni post-hoc test (*P < 0.05) (**b,d**).

We assessed several metabolic parameters of HFD-fed WT mice to investigate the effects of a GCL2505 treatment on HFD-induced metabolic disorders, including insulin resistance and lipid metabolism dysfunction. Oral treatment with GCL2505 decreased body weight gain after 3 weeks administration (Fig. 1a). However, the GCL2505 treatment did not affect energy intake [saline-treated group, 9.40 ± 0.31 kcal/day; GCL2505-treated group, 8.84 ± 0.17 kcal/day (mean ± SE)]. Additionally, the GCL2505 treatment improved glucose tolerance (Fig. 1b,c). Since both the trend of lowered blood glucose levels and the significant decrease in the area under the curve (AUC) during insulin tolerance test were observed in GCL2505-treated mice compared with saline-treated mice, insulin tolerance was also improved by GCL2505 treatment (Fig. 1d,e). The results of the X-ray computed tomography (CT) analysis revealed that the GCL2505 treatment significantly decreased the accumulation of visceral and subcutaneous fat (Fig. 1f,g). To assess fatty acid oxidation, we measured the time-course changes in the relative appearance of ^13^CO_2_ to ^12^CO_2_ (Δ^13^CO_2_/^12^CO_2_) in the expired breath of mice injected with emulsions containing [^13^C] palmitate. The increase in Δ^13^CO_2_/^12^CO_2_ occurred more rapidly for the GCL2505-treated group than for the saline-treated group (Fig. 1h). Moreover, the enrichment of ^13^CO_2_ in the expired breath of GCL2505-treated mice was significantly higher than that of the saline-treated mice according to the AUC (Fig. 1i). These breath test data indicated that the GCL2505 treatment enhanced host systemic fatty acid oxidation.

### Deficiency in GPR43 abolished the anti-metabolic syndrome effect of GCL2505

**Figure 2:**
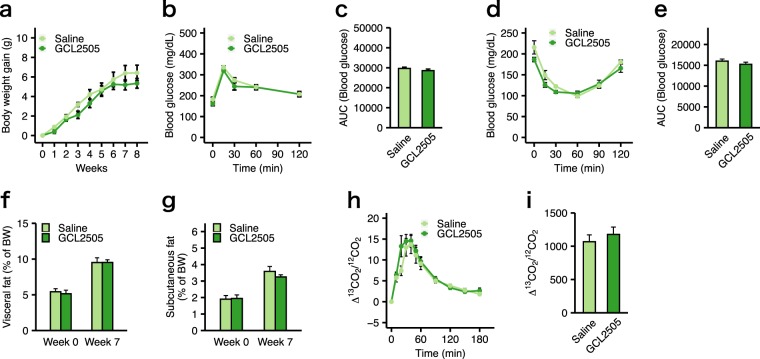
Effects of GCL2505 treatment are not observed in high-fat diet-fed *Gpr43−/−* mice. (**a**) Body weight gain. (**b,c**) Blood glucose curve and the area under the curve (AUC) after an oral glucose challenge. (**d,e**) Blood glucose curve and the AUC after an intraperitoneal injection of insulin. (**f,g**) Computed tomography (CT)-estimated proportions of visceral and subcutaneous fat weight. (**h,i**) Breath ^13^CO_2_:^12^CO_2_ isotope ratio after an oral gavage of ^13^C-labelled palmitate. Specific details are provided in the Fig. 1 legend. Data are presented as the mean ± SEM, and were analysed with Welch’s *t*-test (*P < 0.05) (**a,c,e,f,g,h,i**) or two-way repeated-measurement ANOVA with the Bonferroni post-hoc test (*P < 0.05) (**b,d**).

To clarify the involvement of GPR43 in the effects of GCL2505 on HFD-induced metabolic abnormalities, we investigated the effects of the GCL2505 treatment on the metabolic parameters of *Gpr43−/−* mice. There were no differences in the body weight gain between GCL2505-treated and saline-treated *Gpr43−/−* mice (Fig. 2a). Additionally, the GCL2505 treatment did not influence energy intake [saline-treated group, 9.39 ± 0.35 kcal/day; GCL2505-treated group, 9.18 ± 0.06 kcal/day (mean ± SE)]. In *Gpr43−/−* mice, glucose tolerance (Fig. 2b,c), insulin sensitivity (Fig. 2d,e) and the accumulation of visceral and subcutaneous fat (Fig. 2f,g) were all similar between the GCL2505-treated and saline-treated groups. Moreover, GPR43 deficiency abolished the promotive effects of the GCL2505 treatment on fatty acid metabolism (Fig. 2h,i). These data demonstrated that the GCL2505 treatment supressed insulin resistance and lipid metabolism dysfunction in a GPR43-dependent manner.

### GCL2505 treatment elevated the plasma acetate level, which is inversely associated with body fat accumulation

**Figure 3:**
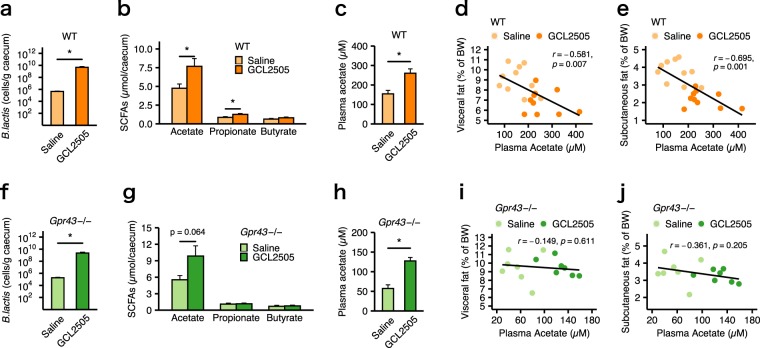
Effects of GCL2505 treatment on the number of *B. lactis* and short-chain fatty acid (SCFA) levels. (**a,f**) Number of *B. lactis* in caecal contents. (**b,g)** Caecal SCFA levels. (**c,h**) Plasma acetate levels. Correlation between plasma acetate levels and either visceral fat **(d,i**) or subcutaneous fat (**e,j**) percentage. Data are from WT mice (**a–e**) and *Gpr43−/−* mice (**f–j**). Data are presented as the mean ± SEM, and were analysed with Welch’s *t*-test to assess the differences between groups [*P < 0.05 (**a–c,f–g**)]. Pearson’s *R* correlation and corresponding *P* values are presented (**d,e,i,j**).

We previously demonstrated that the administration of GCL2505 increases the number of *B. lactis* in the gut and the acetate levels in the caecum and plasma^15^. A quantitative PCR analysis revealed that the number of *B. lactis* in the caecum of WT mice was significantly higher in the GCL2505-treated group than in the saline-treated group (Fig. 3a). The caecal pools of acetate and propionate in WT mice were also higher in the GCL2505-treated group than in the saline-treated group (Fig. 3b). The plasma acetate level of WT mice was also higher in response to the GCL2505 treatment than to the saline treatment (Fig. 3c). Additionally, the plasma acetate level in WT mice was negatively correlated with the amount of either visceral or subcutaneous fat (Fig. 3d,e). Similar to WT mice, the number of *B. lactis* in the caecum of *Gpr43−/−* mice was significantly higher in the GCL2505-treated group than in the saline-treated group (Fig. 3f). The caecal and plasma acetate levels of *Gpr43−/−* mice was considerably higher in the GCL2505-treated group than in the saline-treated group (Fig. 3g,h). However, in *Gpr43−/−* mice, there was no correlation between the plasma acetate level and body fat accumulation (Fig. 3i,j).

### GCL2505 regulated insulin signalling in peripheral tissues in a GPR43-dependent manner

**Figure 4:**
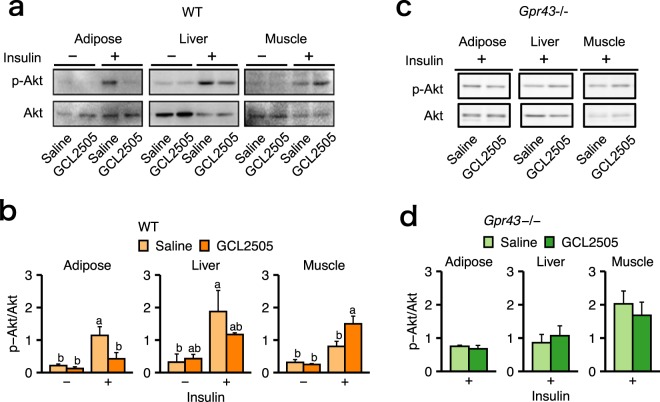
GPR43 suppresses insulin signalling in the adipose tissue, but not in the muscle or liver. Representative images of immunoreactive bands images (**a,c**) and its intensities representing (**b,d**) the insulin-stimulated Akt phosphorylation of Ser473 and total Akt in the adipose tissue, muscle and liver of WT mice (n = 4) and *Gpr43−/−* mice (n = 3) that had fasted for 6 h. Data are presented as the mean ± SEM, and were analysed with Tukey’s test. Different letters next to bars indicate a significant difference (*P* < 0.05).

A previous study confirmed that GPR43 signalling suppresses insulin signalling by suppression of Akt phosphorylation via G(i/o)βγ–PLC–PKC– PTEN in white adipose tissue^10^. Thus, we examined whether GCL2505 treatment affects insulin signalling in peripheral tissues by measuring the phosphorylation level of Akt in several tissues at 5 min after an insulin injection. In WT mice, GCL2505 treatment supressed insulin-induced Akt phosphorylation in the adipose tissue and enhanced phosphorylation in muscle compared with the effects of the saline treatment (Fig. 4a,b). In contrast, the GCL2505 treatment had no effect on the phosphorylated Akt level in the insulin-targeting tissues of *Gpr43−/−* mice (Fig. 4c,d). These data indicated that GCL2505 negatively and positively regulated insulin signalling in the adipose tissue and muscle, respectively, in HFD-fed mice via a GPR43-dependent manner.

## Discussion

*Bifidobacterium animalis* subsp. *lactis* GCL2505 proliferates in the gut and produces SCFAs, especially acetate, which is a GPR43 ligand. We previously revealed that GCL2505 enhances glucose metabolism, while suppressing the accumulation of body fat and adipocyte hypertrophy^15^. In the present study, we demonstrated that GCL2505 suppressed body fat accumulation and enhanced fatty acid/glucose metabolism in WT mice but not in *Gpr43−/−* mice.

A protracted energy imbalance, in which intake exceeds expenditure, triggers the impairment of metabolic homeostasis and the development of metabolic disorders, including obesity and hyperglycaemia. In this study, the daily administration of GCL2505 suppressed the body weight gain, body fat accumulation, and glucose intolerance in HFD-fed mice in the absence of any changes in energy intake. Therefore, GCL2505 may alter host energy expenditure. In fact, the results of the ^13^C-labelled palmitate breath test indicated that the enrichment of ^13^CO_2_ in the expired breath was significantly higher in GCL2505-treated mice than in saline-treated mice, implying that the administration of GCL2505 promotes total-body fatty acid oxidation. Moreover, we previously reported that GCL2505 supresses host adipocyte hypertrophy^15^, which is considered to be a significant predictor of altered blood lipid profiles and glucose–insulin homeostasis^17^. These results suggest that GCL2505 can enhance host systemic energy expenditure, including fatty acid oxidation, resulting in decreased fat accumulation.

Indigestible carbohydrates derived from the diet are fermented by gut microbiota and finally converted to SCFAs such as acetate, propionate and butyrate. These SCFAs are absorbed via the colonic epithelium, and the most abundant SCFA, acetate, reaches concentrations of 19–160 μM in peripheral blood, whereas propionate and butyrate reach 1–13 μM and 1–12 μM, respectively^18^. SCFAs can be incorporated into lipids and glucose, which are the main energy sources for the host^19^. We recently reported that SCFAs also function as signalling molecules and regulate energy homeostasis via GPCRs^10,20^. In the present study, GCL2505 treatment significantly elevated caecal SCFA levels, especially acetate, and plasma acetate levels in both WT and *Gpr43−/−* mice. In contrast, the enhanced fatty acid oxidation and insulin sensitivity induced by the GCL2505 treatment were not observed in *Gpr43−/−* mice. Additionally, plasma acetate level was negatively correlated with the amount of body fat mass in WT mice, but not in *Gpr43−/−* mice. Our findings suggest that the plasma acetate level elevated by GCL2505 activates GPR43, enhances host energy expenditure, and suppresses body fat accumulation.

Insulin signalling in peripheral tissues is associated with the balance of whole-body energy metabolism and storage^21,22^. The insulin tolerance test results for the WT mice indicated that the GCL2505-treated group was more sensitive to insulin than the saline group. However, a previous study confirmed that GCL2505 does not affect insulin secretion^15^, suggesting that GCL2505 can alleviate HFD-induced glucose intolerance by enhancing insulin sensitivity rather than by increasing insulin secretion. Therefore, we investigated whether GCL2505 affects host energy metabolism by modulating insulin signalling in the peripheral tissues. The GCL2505 treatment lowered the phosphorylated Akt level induced by insulin in the adipose tissue of HFD-fed WT mice, but not in that of HFD-fed *Gpr43−/−* mice. Additionally, the insulin-induced Akt phosphorylation was amplified by the GCL2505 treatment in the muscle of HFD-fed WT mice, but not in that of HFD-fed *Gpr43−/−* mice. We previously determined that the oral administration of acetate suppresses adipose insulin signalling^10,23^. Moreover, resistant starch treatments increase SCFA levels and muscle insulin sensitivity^24^. These reports support our findings that plasma acetate levels elevated by GCL2505 activate GPR43 and suppress insulin signalling in adipose tissues, possibly leading to prevention of body fat accumulation and ultimately increased systemic insulin sensitivity and energy utilization in non-adipose tissues.

In the present study, however, we did not consider other factors which could directly affect insulin signalling in the muscle tissue such as GPR43 signalling on the muscle cells or glucagon-like peptide 1 (GLP-1) secreted from the colonic cells. Although the *Gpr43* mRNA in the muscle tissue was reported to be barely expressed compared with the adipose tissue in our previous study^10^, it remains unclear whether elevated acetate levels by probiotics treatment activate muscle GPR43 and the subsequent insulin signalling. Additionally, GLP-1 was reportedly elevated in mice plasma by GCL2505 treatment^15^, and was also reported to restore muscle metabolic insulin activities under insulin-resistance conditions induced by a lipid infusion^25^. Therefore, it is unclear whether other factors affecting the muscle tissue insulin signalling contribute to the probiotics effects, and further study is needed.

**Figure 5:**
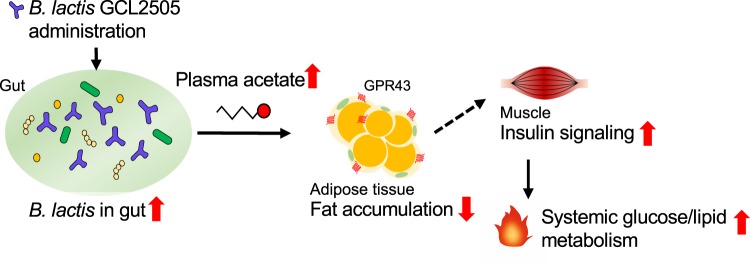
Possible mechanisms underlying the GCL2505 effects on host energy metabolism. Number of *B. lactis* in the gut and the plasma acetate level increase in response to GCL2505. Elevated acetate level modulates host energy metabolism (e.g., suppressed body fat accumulation, increased insulin sensitivity, and enhanced systemic fatty acid metabolism) in a GPR43-dependent manner.

Many probiotics are reported to have beneficial effects on metabolic disorders such as hypertension, obesity, inflammation, glucose homeostasis disorders and abnormal plasma lipid levels^26^. Several probiotic effects on the host have been proposed, such as increased SCFA production, decreased cholesterol levels due to modified bile salt hydrolase activity, increased cell adhesion and mucin production, modulation to the immune system, and interactions with the brain–gut axis via regulated endocrine and neurological functions^27,28^. In the present study, analyses of *Gpr43−/−* mice clearly demonstrated that GCL2505, a highly viable and proliferative probiotic, can promote the production of SCFA in the gut, which function as an essential factor mediating host metabolic homeostasis (e.g., enhanced glucose tolerance and suppressed body fat accumulation) in a GPR43-dependent manner (Fig. 5). Although most probiotic activities are thought to be strain-dependent, the data presented herein suggest that the activation of GPR43 in peripheral tissues is a common mechanism enabling some probiotics increasing gut SCFA levels to modulate host energy metabolism.

## Methods

### Preparation of *Bifidobacterium animalis* subsp. *lactis* GCL2505

*Bifidobacterium animalis* subsp. *lactis* GCL2505 was obtained from Ezaki Glico Co., Ltd. and cultured in Gifu anaerobic medium broth (Nissui) supplemented with up to 1% glucose. For the probiotic treatments, the cultured bacteria were washed and suspended in saline.

### Animal treatments

Five-week-old male C57BL/6J mice were obtained from SLC Inc. The *Gpr43* knockout (*Gpr43−/−*) mice with a C57BL/6 background were generated in a previous study^10^. We confirmed the lack of *Gpr43* mRNA in the adipose tissue and muscle of *Gpr43−/−* mice (see Supplementary Figure). Mice were housed in a controlled environment (12-h light/12-h dark cycle) and fed an HFD (45 kcal% fat; D12451 formula, Research Diets Inc.). After 2 weeks, the mice were divided into control and GCL2505-treated groups (n = 10 wild type, n = 7 *Gpr43−/−*, for each treatment group). The mice in the GCL2505-treated group were orally administered GCL2505 (1 × 10^9^ colony forming units/day) for 6–7 weeks. The mice in the control group were given saline. All experimental procedures involving mice were performed in 2015–2017 according to protocols approved by the Committee on the Ethics of Animal Experiments of the Tokyo University of Agriculture and Technology (Permit No. 28–87) and the Institutional Animal Care and Use Committee of Ezaki Glico Co., Ltd.

### Body fat composition analysis

Body fat composition was analysed as described by Lubura *et al*.^29^, with some modifications. Briefly, mice were anaesthetised by the inhalation of isoflurane and scanned with a LaTheta LCT-100 X-ray CT system (Aloka). Contiguous 1-mm slice images between the proximal end of lumbar vertebra L1 and the distal end of L6 were used for the quantitative assessment completed with LaTheta software (version 2.10, Aloka). Subcutaneous and visceral fat levels were quantified.

### ^13^C-palmitate breath test

To examine total-body fatty acid oxidation, a ^13^C-palmitate breath test was performed for all mice groups after a 7-week treatment. The ^13^C-labelled potassium palmitate was emulsified at 60 °C in 5% lecithin (Wako) dissolved in sterilized water, and 60 μmol/kg was applied by oral gavage in a volume of 75 μl/25 g body weight to mice that had fasted for 4 h. Breath samples were taken at baseline and 15, 30, 45, 60, 75, 90, 105, 120, 150, 180, 210, 240, 270 and 300 min after the treatment with ^13^C-labelled palmitate. To collect ^13^CO_2_ samples at each time-point, animals were placed individually into 120-ml syringes for 60 s. Breath ^13^CO_2_/^12^CO_2_ enrichments were analysed by infrared spectrometry with an Otsuka POCone system (Otsuka Electronics, Co., Ltd., Japan), and the Δ^13^CO_2_/^12^CO_2_ ratio was expressed as differences from baseline.

### Oral glucose tolerance test

Mice were fasted for 6 h and injected with glucose by gavage (2 g/kg glucose). The glucose content of blood collected from the tip of the tail vein was determined with the Glucose Pilot glucose meter (Aventir Biotech).

### Insulin tolerance test

Mice were fasted for 3 h and injected with insulin (Sigma) intraperitoneally (1 mU/g body weight). The glucose content of blood collected from the tip of the tail vein was determined with the Glucose Pilot glucose meter.

### Assay of biochemical responses to insulin

Mice were injected with insulin (0.15 U/kg, Sigma) intraperitoneally. After 5 min, liver, skeletal muscle and white adipose tissues were dissected and immediately homogenised in lysis buffer (TNE buffer with 10% NP-40, aprotinin and a phosphatase inhibitor). An immunoblot analysis was performed as described^30^. Full length blot images are shown in Supplementary Information.

### Biochemical analysis

Plasma and caecal acetate levels were enzymatically measured in duplicate with a commercial kit (Acetate Colorimetric Assay Kit, Sigma-Aldrich). The analysis of other caecal SCFAs was performed as follows. Caecal samples were suspended in 5% (w/v) metaphosphate-PBS (49 ml/g sample). Suspensions were subsequently filtered with a 3-kDa MWCO spin column (Pall Corporation) and analysed with a GC-2014 gas chromatography-flame ionisation detector system (Shimadzu) equipped with a glass column packed with 60/80 Shincarbon A coated with Themon-3000 (Shinwa-kakou).

### Quantitative PCR analysis of the number of *B. lactis* in the caecum

Bacterial DNA was extracted from the caecum contents as previously described^13^. A quantitative PCR assay was performed to calculate the number of *B. lactis*. The PCR program was as follows: annealing at 60 °C for 20 s and extension at 72 °C for 50 s. The sequences of the *B. lactis*-specific primers were 5′-CCCTTTCCACGGGTCCC-3′ (forward) and 5′-AAGGGAAACCGTGTCTCCAC-3′ (reverse).

### Statistical analysis

Data are herein presented as the mean ± SEM. All statistical analyses were conducted with the open-source software program R (version 3.4.2) (http://cran.r-project/org). A *P* value < 0.05 was used as the threshold for determining significant differences.

## Supplementary information

Supplementary information.

## Data Availability

The datasets generated during and/or analysed during the current study are available from the corresponding author on reasonable request.
