# Supplementary material for: Bifidobacterium animalis subsp. lactis GCL2505 modulates host energy metabolism via the short-chain fatty acid receptor GPR43
Source: Sci Rep. 2020 Mar 5;10:4158. doi: 10.1038/s41598-020-60984-6 (PMC7057994; doi:10.1038/s41598-020-60984-6)

Online Supporting Materials

Bifidobacterium animalis subsp. *lactis* GCL2505 modulates host energy metabolism via the short-chain fatty acid receptor GPR43.

Authors

Hiroko Horiuchi[1], Kohei Kamikado[1], Ryo Aoki[1], Natsuki Suganuma[1], Tomohiko Nishijima[1], Akiho Nakatani[2] and Ikuo Kimura[2]

Author Affiliation

[1]Institute of Health Sciences, Ezaki Glico Co., Ltd., Nishiyodogawa, Osaka 555-8502, Japan

[2]Department of Applied Biological Science, Graduate School of Agriculture, Tokyo University of Agriculture and Technology, Tokyo 183-8509, Japan

Online Supporting Materials

Methods

Analysis of mRNA expression of GPR43 gene

Total RNA was immediately extracted from frozen muscle and epididymal adipose tissue segments via the QuickGene RNA tissue Kit SII (RT-s2; KURABO). Complementary DNA was transcribed using RNA templates with High-Capacity cDNA Reverse Transcription Kit (Termo Fisher Scientific). cDNA samples of GPR43 genes (*Ffar2*, Free Fatty Acid Receptor 2) and ribosomal protein S18 genes (*Rps18*) were amplified by the CFX96 Real-Time PCR Detection System (Bio-Rad) with PrimeTime qPCR Assays (Integrated DNA Technologies). PCR were performed as follows; annealing at 60°C for 20 s and extension at 72°C for 50 s. Sequences of primers are as follows; 5′- TTCTTACTGGGCTCCCTGCC-3′ (forward), 5′- TACCAGCGGAAGTTGGATGC-3′ (reverse), and 5′-（FAM)AAGTCCGCC(ZEN)AGGGTCAGATTAAGC(IBFQ) -3′ (probe) for *Ffar2*; 5′- ACACCACATGAGCATATCTCC-3′ (forward), 5′-CCTGAGAAGTTCCAGCACAT-3′ (reverse), and 5′-AGCCTTCGC(ZEN)CATCACTGCCATTA(IBFQ)-3′ (probe) for *Rps18*. Expression was quantified in duplicate. The *Ffar2* mRNA levels were normalized using *Rps18* as an internal standard.

Online Supporting Materials

Supplementary Figure. The mRNA level of GPR43 (*Ffar2* gene) in adipose tissue (left) and muscle (right) of wild type (WT) and GPR43-knock out (KO) mice. Data represent the mean ± SEM. Statistical analyses were performed using Welch's *t*-test.

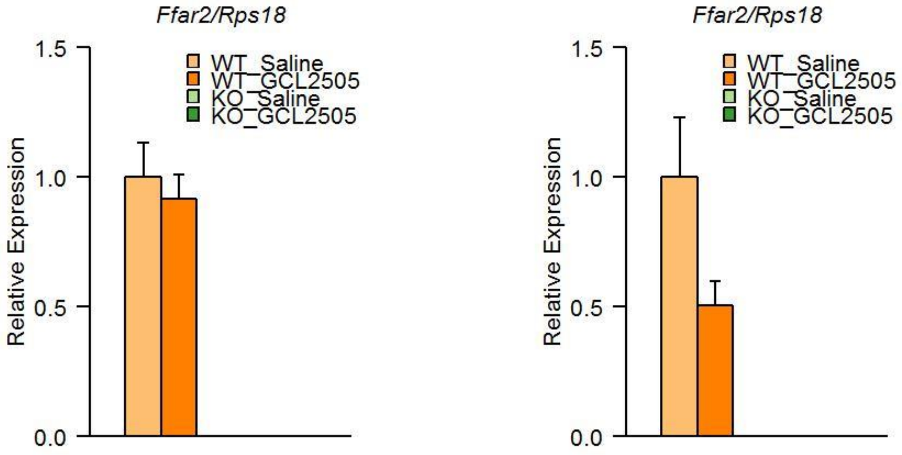

Online Supporting Materials

Supplementary Information 1. Full length images of Figure 4a representing Akt phosphorylation of Ser473 (a) and total Akt (b) in the adipose tissue (Lane A1–4), liver (Lane L1–4) and muscle (Lane M1–4) of WT mice. Lane No. 1, insulin−, saline group; 2, insulin−, GCL2505 group; 3, insulin+, saline group; 4, insulin+, GCL2505 group.

a

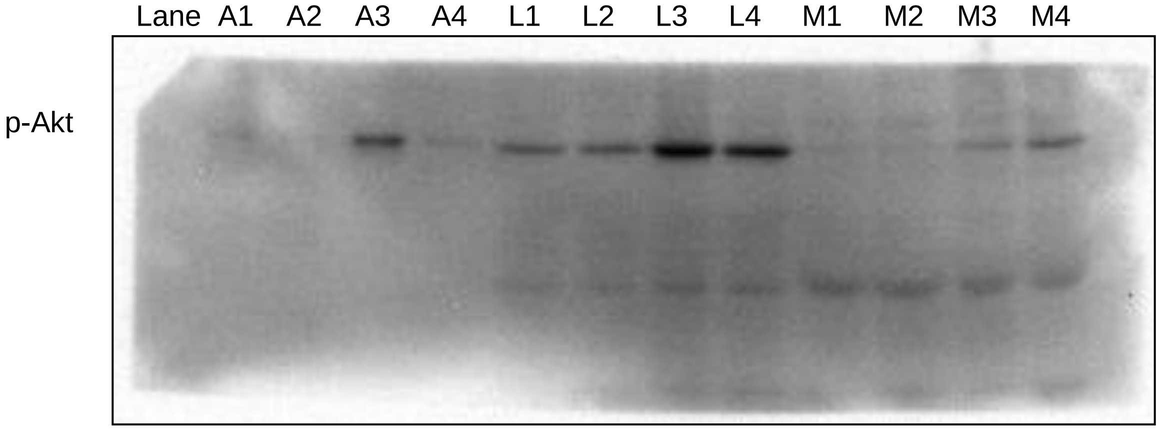

b

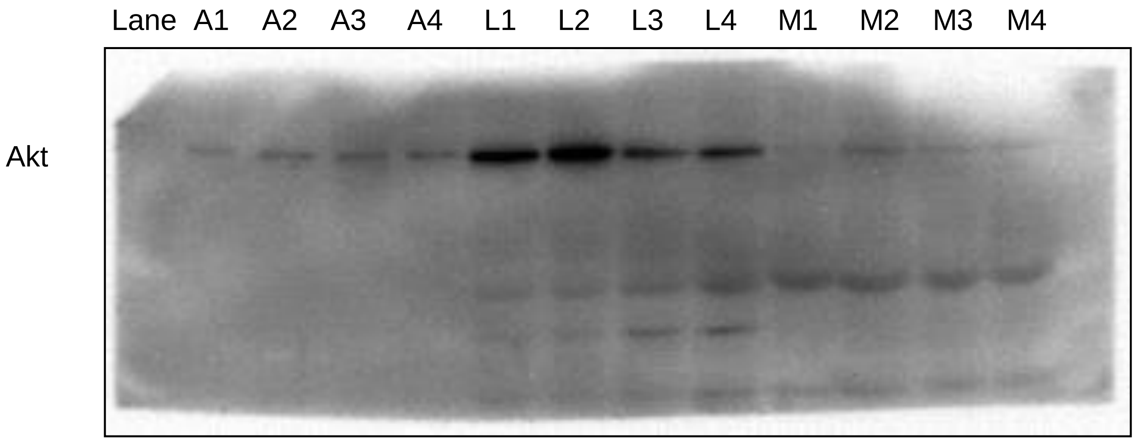

Online Supporting Materials

Supplementary Information 2. Full length images of Figure 4c representing Akt phosphorylation of Ser473 (a, c) and total Akt (b, d) in the adipose tissue (a, b), liver and muscle (c, d) of *Gpr43−/−* mice. Lane No. 1, 3, 5, insulin+, saline group; No. 2, 4, 6, insulin+, GCL2505 group.

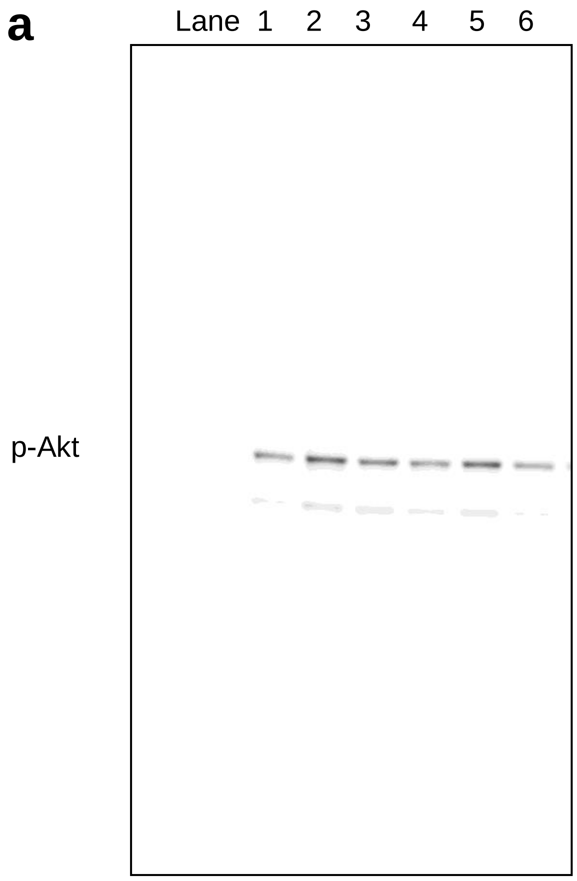

Online Supporting Materials

b

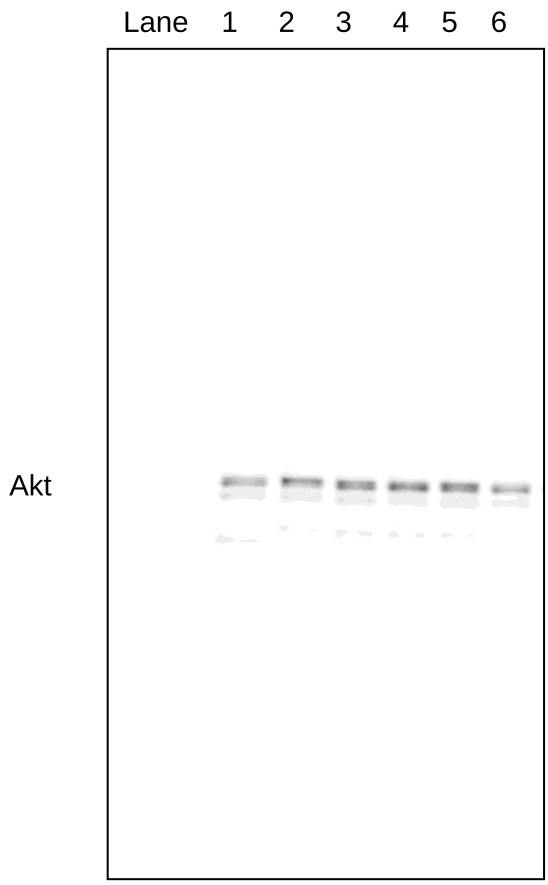

c

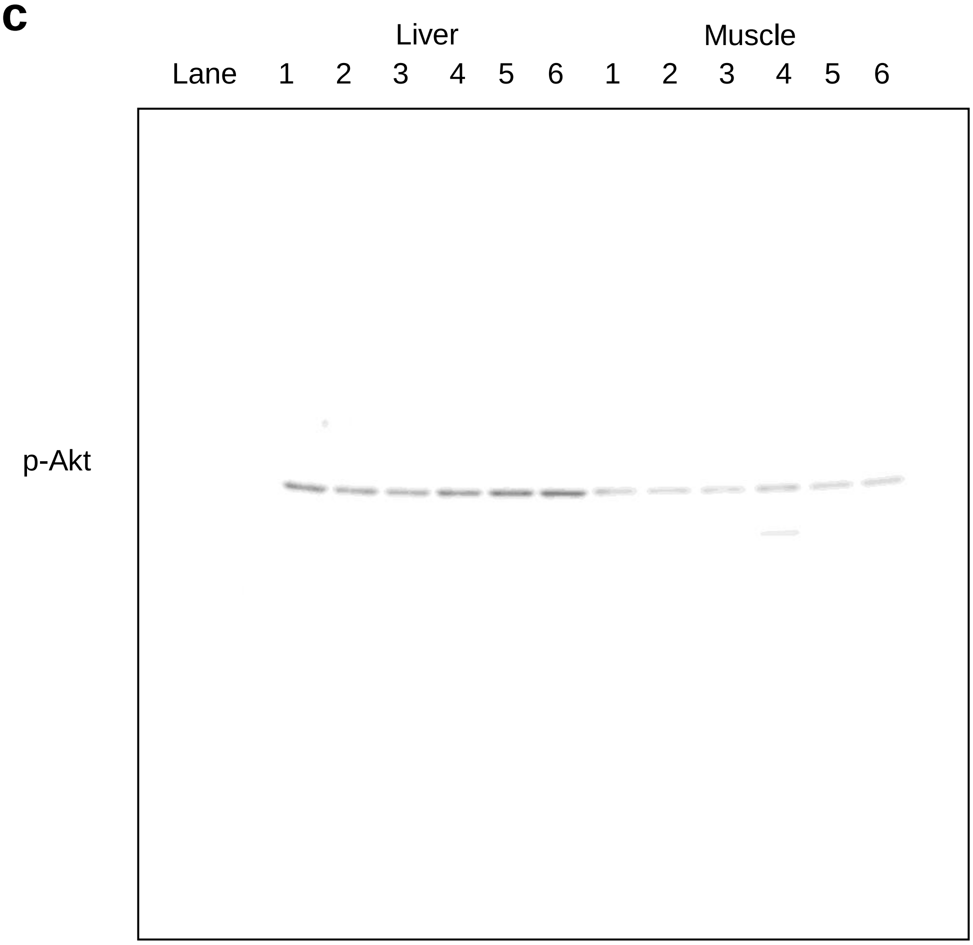

d

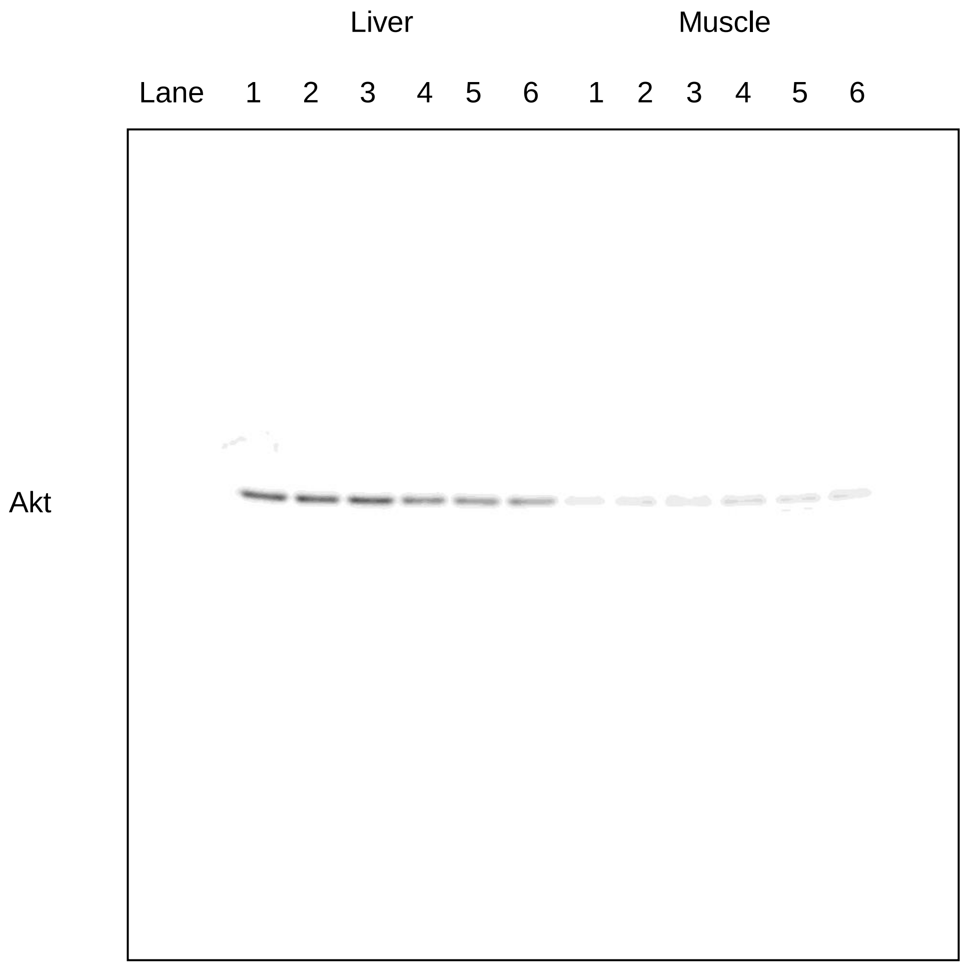

Supplement: Supplementary file 1 — Supplementary information. [file 41598_2020_60984_MOESM1_ESM.pdf]
